# Intermuscular coherence of plantar and dorsiflexor muscles in older adults with Parkinson’s disease and age-matched controls during bipedal and unipedal stance

**DOI:** 10.3389/fnagi.2023.1093295

**Published:** 2023-02-20

**Authors:** Rowan R. Smart, Anis Toumi, Owen D. Harris, Sylvain Cremoux, Brian H. Dalton, Daryl J. Wile, Jennifer M. Jakobi

**Affiliations:** ^1^School of Health and Exercise Sciences, University of British Columbia Okanagan, Kelowna, BC, Canada; ^2^Centre de Recherche Cerveau et Cognition, UMR CNRS, Université Paul Sabatier Toulouse III, Toulouse, France; ^3^Southern Medical Program, Centre for Chronic Disease Prevention and Management, University of British Columbia Okanagan, Kelowna, BC, Canada

**Keywords:** balance, posture, sway, electromyography, center of pressure, older adults, aging, single-leg

## Abstract

**Introduction:**

Postural instability increases with age and is exacerbated in neurological disorders such as Parkinson’s disease (PD). Reducing the base of support from bipedal to unipedal stance increases center of pressure (CoP) parameters and intermuscular coherence in lower-leg muscles of healthy older adults. To further develop an understanding of postural control in an altered state of neurological impairment, we explored intermuscular coherence in lower-leg muscles and CoP displacement in older adults with PD.

**Methods:**

This study measured surface EMG from the medial (MG) and lateral (LG) gastrocnemii, soleus (SOL), and tibialis anterior (TA), and examined EMG amplitude and intermuscular coherence during bipedal and unipedal stance on a force plate with firm (no foam) and compliant (standing on foam) surface conditions in nine older adults with PD (70±5 years, 6 females) and 8 age-matched non-Parkinsonian older adults (5 females). Intermuscular coherence was analyzed between agonist-agonist and agonist-antagonist muscle pairs in the alpha (8-13 Hz) and beta (15-35 Hz) frequency bands.

**Results:**

CoP parameters increased from bipedal to unipedal stance in both groups (*p* < 0.01), but did not increase from the firm to compliant surface condition (*p* > 0.05). During unipedal stance, CoP path length was shorter in older adults with PD (2027.9 ± 1074.1 mm) compared to controls (3128.5 ± 1198.7 mm) (*p* < 0.01). Alpha and beta agonist-agonist and agonist-antagonist coherence increased by 28% from bipedal to unipedal stance (*p* > 0.05), but did not differ between older adults with PD (0.09 ± 0.07) and controls (0.08 ± 0.05) (*p* > 0.05). The older adults with PD also had greater normalized EMG amplitude of the LG (63.5 ± 31.7%) and TA (60.6 ± 38.4%) during the balance tasks (*p* > 0.05) than the non-Parkinsonian counterparts.

**Discussion:**

Older adults with PD had shorter path lengths during unipedal stance and required greater muscle activation than older adults without PD to perform the tasks, but intermuscular coherence did not differ between the groups. This may be attributable to their early disease stage and high motor function.

## 1. Introduction

Postural instability contributes to fall risk and injury in older adults with Parkinson’s disease (PD; Jankovic, 2008). Postural instability can be subjectively assessed as part of a neurological exam, and can be quantified in the laboratory by measuring fluctuations in the center of pressure (CoP) during standing. Prior studies have reported greater CoP area and CoP variability in the mediolateral direction in older adults with PD compared to older adults without PD during bipedal stance (Mitchell et al., 1995; Błaszczyk et al., 2007; Chastan et al., 2008). Standing balance can be further challenged by progressing from bipedal to unipedal stance (Ringhof et al., 2015; Watanabe et al., 2018), or by increasing the compliance of the standing surface through adding a layer of foam, which, in turn, reduces the reliability of somatosensory cues from the feet (Baudry et al., 2014; Papegaaij et al., 2014; Gosselin and Fagan, 2015).

Maintaining upright balance during unipedal compared to bipedal stance requires greater activation of the plantar flexors and results in greater intermuscular coherence (García-Massó et al., 2016; Watanabe et al., 2018). Intermuscular coherence represents common synaptic drive to pairs of muscles working together, or to opposing muscles acting around the same joint, and represents the shared variance (correlation) between each muscle’s electromyography (EMG) frequency components (Halliday et al., 2003). This common drive can be evaluated in bandwidths of interest such as the alpha band (8–13 Hz), which is suggested to represent inputs from stretch-reflex system, motor cortex, brainstem and cerebellum (Laine and Valero-Cuevas, 2020), and the beta band (15–35 Hz) representing cortical inputs (Conway et al., 1995; Grosse et al., 2002). Watanabe et al. (2018) reported that beta band coherence between the medial gastrocnemius (MG), lateral gastrocnemius (LG), and soleus (SOL) muscles was greater during unipedal compared to bipedal stance in young and old adults, likely as a result of increased cortical demands, and was also correlated with increased CoP standard deviation (SD) and CoP displacement velocity in both the anteroposterior and mediolateral directions. The increased difficulty of the unipedal stance likely requires greater cortical contribution, and input from other systems involved in balance. This would lend to an increase in alpha and beta band intermuscular coherence compared with bipedal stance.

Intermuscular coherence may be a useful marker of neural control of voluntary muscle activity, and appears to be altered in older adults with PD. When treated with dopaminergic medication, intermuscular coherence in older adults with PD is greater, and isometric force steadiness is lower for the upper (Laine and Valero-Cuevas, 2020) and lower limbs (Flood et al., 2019). Deep brain stimulation (DBS) in the subthalamic nucleus (STN) is effective in treating the motor symptoms of PD, and Marsden et al. (2001) showed that STN DBS in the absence of medication increased upper limb intermuscular coherence, toward that seen in non-Parkinsonian older adults. PD treatments including dopaminergic medication and DBS may increase intermuscular coherence by normalizing central coordination of muscle activity. To our knowledge, intermuscular coherence has not been studied in lower limb muscles across a range of balance tasks when postural stability is challenged in older adults with PD. With prior studies demonstrating increased coherence coupled with increased variability of isometric force output (Flood et al., 2019; Laine and Valero-Cuevas, 2020), one could expect that increased coherence may also be contributing to increased sway observed in older adults with PD (Mitchell et al., 1995; Błaszczyk et al., 2007; Chastan et al., 2008; Kamieniarz et al., 2021).

We examined plantar and dorsiflexor intermuscular coherence and CoP parameters during quiet (e.g., bipedal stance on a firm surface) and challenging (e.g., unipedal stance on a compliant surface) standing balance tasks in older adults with PD tested during the ON-phase of their levodopa medication cycle, and in non-Parkinsonian older adults. We hypothesized that in testing older adults with PD during the ON-phase of their medication cycle, they would have greater CoP displacements and intermuscular coherence compared to age-matched older adults without PD, and that this group difference would be accentuated in the challenging balance condition with unipedal stance on a compliant surface.

## 2. Materials and methods

### 2.1. Participants

Nine older adults with PD (*n* = 6 females) and 8 age-matched older adults with no known neurological conditions (*n* = 5 females) serving as a control group were recruited from local PD society support groups and the local community. Exclusion criteria included the presence of lower-limb injuries in the prior 6 months, neurological conditions unrelated to PD, coexisting neuropathy, or muscular, metabolic, or cardiovascular conditions that could affect standing balance. All PD participants were treated with dopaminergic medication and were tested when they perceived their medication to be effective, typically 1.5 h after medication intake. Beyond the prescribed dosages of levodopa for the older adults with PD, none of the participants reported taking other medications that may affect balance. The study was approved by the Institutional Clinical Research Ethics Board (H16-02963) and all participants provided written informed consent prior to participating.

### 2.2. Experimental setup

The skin was exfoliated and cleaned with 70% isopropyl alcohol and Ag–AgCl cloth electrodes (Kendall^™^ H59P, Covidien, Mansfield, MA, United States) were placed on the skin surface of the MG, LG, SOL, and tibialis anterior (TA) according to surface electromyography non-invasive assessment of muscles (SENIAM) guidelines (Hermens et al., 2000), with an interelectrode distance of 2 cm. The EMG signals were amplified (100 ×) and band-pass filtered (8–150 Hz) using a Coulbourn instrument unit (Allentown, Pennsylvania, PA, United States), sampled at 1,000 Hz, and converted from analog to digital using the Power 1401 (CED, Cambridge, England). Ground electrodes were placed on bony prominences of the femur and tibia.

To obtain EMG measures of maximal muscle activation for the plantar flexors (MG, LG, and SOL) and dorsiflexors (TA), isometric maximal voluntary contractions (MVC) were performed with participants seated in a commercially available dynamometer (System 4 PRO^™^, Biodex Medical Systems, Shirley, NY, United States) with the ankle of the more affected leg (PD) or dominant leg (older adults without PD) secured to the dynamometer footplate with inelastic straps while the other leg rested on a foot rest. Participants sat with hips at 95° and the knee of the tested leg extended to ~ 160° (180° being terminal knee extension). The foot was secured to the dynamometer with the lateral malleolus aligned with the axis of rotation of the dynamometer and the ankle positioned at the participant’s standing ankle angle (~ 96° plantar flexion), measured at the medial malleolus as the angle between the tibia and first metatarsal, with 90° being a neutral ankle angle. The lateral malleolus of the tested leg was aligned with the axis of rotation of the dynamometer. Torque (Nm) was sampled from the Biodex at 1,000 Hz using a 16-bit Power 1401 (Cambridge Electronic Design (CED), Cambridge, England), stored for offline analysis using Spike 2 v7.12 (CED), and subsequently converted offline to Newtons (N) of force using the lever arm length of the footplate. Visual feedback of the torque signal was provided in real-time using a 52 cm monitor positioned 1 m in front of participants at eye level.

Standing balance tasks were performed on the rigid force plate (Length: 46.4 cm, Width: 50.8 cm, Height: 10.2 cm; Advanced Medical Technology, Inc., Watertown, Model OR6–5, Newton, MA, United States) under two conditions (firm and compliant). The firm surface consisted of the force plate alone, and the compliant surface consisted of a foam pad (10.2 cm thick; density, 0.016 g/cm^3^) placed on the force plate, with dimensions equivalent to the area of the force plate. A full-body safety harness was worn for all trials in the event of a loss of balance. Signals from the force plate were converted from analog to digital using a 16-bit Power 1401 (CED, Cambridge, England) at a sampling frequency of 1,000 Hz.

### 2.3. Experimental protocol

Upon arrival to the laboratory, the motor examination portion of the Movement Disorders Society Unified Parkinson’s Disease Rating Scale (MDS-UPDRS) was conducted for each of the PD participants by a member of the research team who was trained by a neurologist to conduct the tests. Medication use and personal characteristics (e.g., age, height, and weight) were documented. Subsequently, both groups performed isometric plantar flexion and dorsiflexion MVCs in the Biodex dynamometer, and then executed the standing balance tasks. Three isometric plantar flexion MVCs were undertaken with ~ 2–3 min rest between each contraction, and the highest force MVC was used for the measure of maximal force and maximal muscle activity (surface EMG) for the plantar flexors (MG, LG, and SOL). Participants were instructed to push the ball and toes of their foot as hard and as fast as they could against the footplate for 5 s. Three dorsiflexion MVCs were then performed to obtain maximal EMG of the TA, requiring participants to pull their toes toward themselves as hard as they could for 5 s. These maximal EMG values obtained during MVCs were used to normalize the EMG from the balance tasks.

During bipedal stance, participants stood comfortably with their feet shoulder width apart, whereas the unipedal stance required PD participants to stand on the more affected leg, and the older adults without PD to stand on their dominant leg. The knee of the other leg was flexed to elevate the foot off the surface of the force plate. During the balance tasks, participants folded their arms across their chest and kept their eyes open with their gaze fixed on the wall in front of them. The order of bipedal and unipedal stance, as well as the order of the two conditions (firm and compliant surface), was randomized. Participants performed each trial for 60 s, and if they were unable to perform the trial for the full 60 s, a second trial was conducted. The trial with the longest duration was used for analyses to obtain a measure of sway that represented the participant’s best performance of the task.

### 2.4. Data analysis

#### 2.4.1. Center of pressure parameters

**Figure 1:**
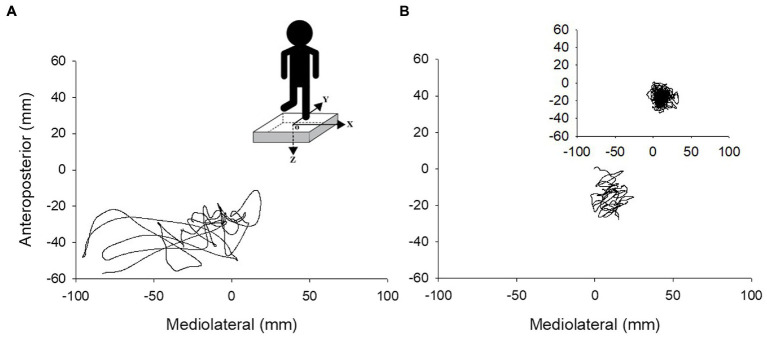
Center of pressure traces during 10 s of unipedal stance for an older adult with PD **(A)** and older adult without PD **(B)**. Insert in A shows the force plate with the *x*, *y*, and *z* axes of force and the center of the force plate (o), and the insert in B depicts a complete 60 s trial for one older adult without PD.

The force plate signals were filtered digitally with a 10 Hz low-pass filter (second order Butterworth). The force plate provided three forces and moment components: The anteroposterior axis was positive in the anterior direction, the mediolateral axis was positive toward the right side, while the vertical axis was positive when directed downward (Figure 1). CoP area corresponded to the surface area of an ellipse that covered 95% of the CoP displacement (Paillard and Noé, 2015). To account for differences in trial duration between individual participants, CoP area was normalized to each participant’s trial duration in seconds (s). The CoP amplitude was calculated as the distance between the minimum and maximum displacement in both anteroposterior (CoP *y*) and mediolateral (CoP *x*) axes. CoP path length was calculated as the sum of the CoP displacement in the mediolateral and anteroposterior axes. The CoP displacement velocity (mm/s) was calculated by dividing the total path length (mm) of the trial by the trial duration (s; Luoto et al., 1998). Velocity was calculated using the combined path length of the anteroposterior and medio-lateral CoP components.

#### 2.4.2. Electromyography

To obtain a linear envelope for EMG amplitude measures (Shiavi et al., 1998), EMG signals were further digitally band-pass filtered between 10 and 400 Hz, full-wave rectified, and low pass filtered at 9 Hz using 4th order, zero-lag Butterworth filters with custom MATLAB scripts (Desmyttere et al., 2018). The envelope was equivalent to the maximal duration of the balance tasks. Using the digitally filtered signal, we quantified EMG as the mean root mean squared (RMS) amplitude of the signal over the entire duration of the trial, and EMG of the individual muscles during the balance tasks were normalized to their maximal values over 1 s at the peak of the isometric plantar flexion (LG, MG, and SOL) and dorsiflexion (TA) MVCs. Coactivation ratio was calculated by dividing the normalized EMG of the TA by the sum of the normalized plantar flexors’ EMG.

**Figure 2:**
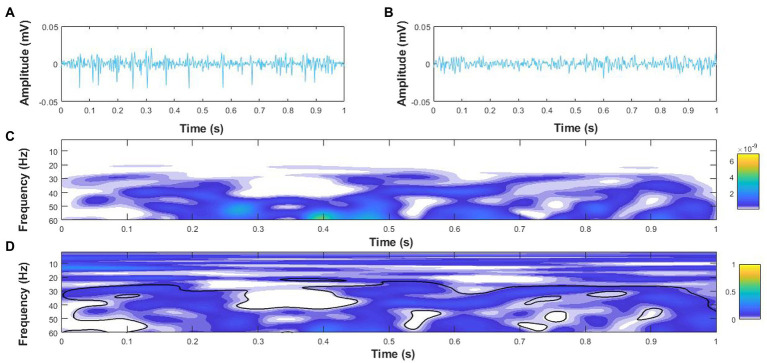
Example coherence analysis for a 1 s epoch of surface electromyography (EMG) signals for the medial gastrocnemius (MG), **(A)** and soleus **(B)** of an older adult with PD during single leg stance. **(C)** Wavelet cross-spectrum of the MG and soleus EMG signals, and **(D)** Wavelet-magnitude squared coherence between MG and soleus EMG in the time-frequency domains with the black outline denoting regions with significant coherence. The yellow and blue scales on the right-hand side represent the power of the cross-spectrum **(C)** and magnitude-squared coherence **(D)**, respectively.

Intermuscular coherence was calculated in the time-frequency domain for agonist–agonist and agonist–antagonist muscle pairs using the hardware filtered EMG signal (Coulbourn; Allentown, Pennsylvania, PA, United States) before applying the digital processing in MATLAB. Pooled agonist–agonist coherence was considered for each participant as the mean coherence across LG–MG, LG–SOL, and MG–SOL muscle pairs, while pooled agonist–antagonist coherence was considered as the mean coherence across MG–TA, LG–TA, and SOL–TA muscle pairs (Desmyttere et al., 2018). Coherence was analyzed in 1-s epochs across the entire trial duration. The auto-spectrum of each EMG signal and the cross-spectrum between each EMG signal combination were first quantified using the WavCrossSpec software for wavelet coherence analysis (Grinsted et al., 2004) with a frequency resolution of 2.57–257.02 Hz in 0.3640 Hz steps. The magnitude-squared cross-spectrum was then normalized by the product of the two EMG auto-spectra to obtain the magnitude-squared coherence between each signal. Intermuscular interactions were defined as the volume under magnitude-squared coherence values in alpha (8–13 Hz) and beta (15–35 Hz) frequency bands (Elie et al., 2021) where the correlation between EMG time-series was significant on the wavelet cross-spectrum (Bigot et al., 2011; Figure 2).

### 2.5. Statistical analysis

Statistical analyses were conducted using Statistical Package for Social Sciences (SPSS) version 28 (IBM, Armonk, NY, United States). Participant characteristics of age, height, and mass were compared between older adults with PD and older adults without PD using independent samples T-tests. The majority of repeated factor variables were normally distributed and thus parametric statistics were used for comparisons. The CoP parameters, normalized EMG of each muscle, coactivation ratio, and intermuscular coherence for the agonist–agonist and agonist–antagonist muscle pairs in the alpha (8–13 Hz) and beta (15–35 Hz) bands were analyzed using a 2 (group: PD and older adults without PD) × 2 (stance: bipedal and unipedal) × 2 (condition: firm and compliant surface) mixed-model analysis of variance (ANOVA) with post-hoc tests corrected for multiple comparisons. For the mixed-model ANOVAs, when the assumption of sphericity was violated according to Mauchly’s test of sphericity (*p* ≤ 0.05), degrees of freedom were corrected using Greenhouse–Geisser estimates. For significant interactions, post-hoc tests were conducted between groups using independent samples *T*-tests and corrected for multiple comparisons. Values were reported as mean ± SD in text and figures.

## 3. Results

### 3.1. Subject characteristics

Older adults with PD and older adults without PD did not differ in age, height, mass, or standing ankle angle (*p* > 0.05), although the older adults without PD were stronger than older adults with PD during plantar flexion (*p* = 0.05; Table 1).

**Table 1:** Subject characteristics.

	Sex (M/F)	Age (years)	Height (cm)	Mass (kg)	PD duration (years)	UPDRS motor score	Standing ankle angle (°)	MVC (*N*)
PD	3/6	70 ± 5	166 ± 9	66 ± 10	6 ± 2	13 ± 6	93 ± 8	319 ± 99^*^
OA	3/5	71 ± 6	163 ± 7	67 ± 16	NA	NA	98 ± 4	430 ± 178

PD, older adults with Parkinson’s disease; OA, older adults without PD; M, male; F, female; °, degree; N, Newtons, NA, not applicable. Standing ankle angle was measured as the plantar flexion angle, with 90° being neutral. ^*^, differs from OA (*p* < 0.05).

### 3.2. Center of pressure parameters

**Figure 3:**
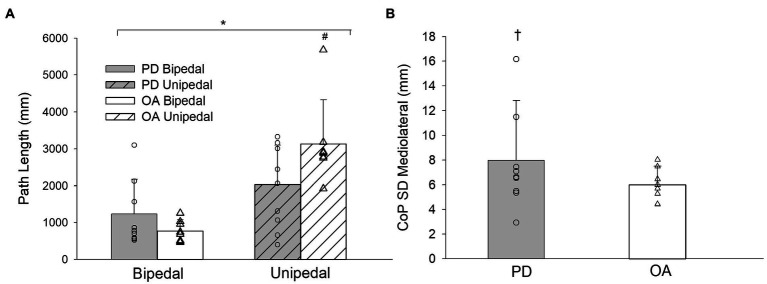
**(A)** Path length for older adults with PD and older adults without PD during bipedal and unipedal stance collapsed across conditions. **(B)** center of pressure (CoP) standard deviation (SD) mediolateral for older adults with PD and older adults without PD collapsed across conditions. PD, older adults with Parkinson’s disease; OA, older adults without PD. ^*^, unipedal greater than bipedal; ^#^, greater in older adults without PD during unipedal stance. ^†^, group main effect for CoP SD Mediolateral (*p* = 0.06).

There were no significant stance by group by condition interactions for the CoP parameters and trial duration (Table 2). F statistics and effect sizes (*η*_p_^2^) are provided for two-way interactions and stance main effects of the CoP parameters in Table 3. The stance by group and stance by condition interactions were not significant for postural sway parameters of trial duration, CoP SD in the anteroposterior and mediolateral directions, CoP displacement amplitude in the anteroposterior and mediolateral directions, CoP area, and time-normalized CoP area. The stance by group interaction was significant for path length and CoP displacement velocity. Path length increased from bipedal to unipedal stance, and was greater in older adults without PD than older adults with PD during unipedal stance (*p* = 0.009), while CoP displacement velocity increased from bipedal to unipedal stance and the interaction occurred as the increase was greater in older adults without PD than older adults with PD (Figure 3A).

**Table 2:** Center of pressure (CoP) parameters for older adults with Parkinson’s disease and older adults without PD in bipedal and unipedal stances during firm and compliant surface conditions.

Firm	CoP area (mm^2^)	Norm. area (mm^2^/s)	CoP velocity (mm/s)	CoP SD AP (mm)	CoP amplitude AP (mm)	CoP amplitude ML (mm)
Bipedal PD	545.3 ± 467.0	9.1 ± 7.9	18.8 ± 14.9	5.5 ± 1.9	34.1 ± 12.6	26.0 ± 13.8
Bipedal OA	311.9 ± 202.1	5.2 ± 3.4	11.8 ± 4.5	5.4 ± 1.9	29.1 ± 8.2	17.3 ± 8.3
Unipedal PD	1578.7 ± 573.2^*^	83.5 ± 92.5^*^	58.9 ± 19.5^*^	10.3 ± 2.5^*^	55.5 ± 15.4^*^	47.3 ± 27.8^*^
Unipedal OA	1789.7 ± 880.9^*^	41.8 ± 34.6^*^	69.5 ± 26.1^*^	10.9 ± 3.8^*^	67.6 ± 25.9^*^	44.1 ± 9.3^*^
*Compliant*						
Bipedal PD	540.8 ± 265.0	8.1 ± 4.3	22.7 ± 17.4	7.7 ± 3.0	40.6 ± 13.4	27.2 ± 8.8
Bipedal OA	437.4 ± 281.9	7.3 ± 4.7	13.9 ± 5.6	6.5 ± 2.2	35.4 ± 10.9	20.7 ± 7.8
Unipedal PD	1856.0 ± 829.2^*^	51.2 ± 53.6^*^	50.2 ± 17.1^*^	11.7 ± 2.6^*^	68.3 ± 17.7^*^	51.8 ± 40.4^*^
Unipedal OA	1575.0 ± 521.1^*^	45.6 ± 34.2^*^	65.0 ± 20.0^*^	9.9 ± 2.0^*^	81.6 ± 55.0^*^	52.6 ± 19.5^*^

BP, bipedal; UP, unipedal; PD, older adults with Parkinson’s disease; OA, older adults without PD; CoP, center of pressure; SD, standard deviation; Norm. area, time-normalized area. AP, anteroposterior; ML, Mediolateral; s, seconds. ^*^Differs from bipedal stance for same surface condition.

**Table 3:** Statistical interactions and main effects for CoP parameters.

	Stance * Group	Stance * Condition	Stance main effect
Trial duration	*F*_(1,29)_ = 1.7, *η*_p_^2^ = 0.06, *p* = 0.2	*F*_(1,29)_ = 0.03, *η*_p_^2^ = 0.001, *p* = 0.9	*F*_(1,29)_ = 18.3, *η*_p_^2^ = 0.4, *p* < 0.001
CoP area	*F*_(1,26)_ = 0.3, *η*_p_^2^ = 0.01, *p* = 0.9	*F*_(1,26)_ = 0.01, *η*_p_^2^ = 0.001, *p* = 0.9	*F*_(1,26)_ = 101.3, *η*_p_^2^ = 0.8, *p* < 0.001
Norm. Area	*F*_(1,25)_ = 1.0, *η*_p_^2^ = 0.04, *p* = 0.3	*F*_(1,25)_ = 0.5, *η*_p_^2^ = 0.02, *p* = 0.5	*F*_(1,25)_ = 19.5, *η*_p_^2^ = 0.4, *p* < 0.001
CoP velocity	*F*_(1,29)_ = 9.4, *η*_p_^2^ = 0.2, *p* = 0.005	*F*_(1,29)_ = 2.1, *η*_p_^2^ = 0.07, *p* = 0.2	*F*_(1,29)_ = 173.1, *η*_p_^2^ = 0.9, *p* < 0.001
CoP SD AP	*F*_(1,29)_ = 0.00, *η*_p_^2^ = 0.00, *p* = 0.9	*F*_(1,29)_ = 1.4, *η*_p_^2^ = 0.05, *p* = 0.2	*F*_(1,29)_ = 55.8, *η*_p_^2^ = 0.7, *p* < 0.001
CoP SD ML	*F*_(1,29)_ = 0.00, *η*_p_^2^ = 0.00, *p* = 0.99	*F*_(1,29)_ = 0.02, *η*_p_^2^ = 0.001, *p* = 0.9	*F*_(1,29)_ = 30.0, *η*_p_^2^ = 0.5, *p* < 0.001
CoP amplitude AP	*F*_(1,29)_ = 2.0, *η*_p_^2^ = 0.06, *p* = 0.2	*F*_(1,29)_ = 0.3, *η*_p_^2^ = 0.01, *p* = 0.6	*F*_(1,29)_ = 27.8, *η*_p_^2^ = 0.5, *p* < 0.001
CoP amplitude ML	*F*_(1,29)_ = 0.4, *η*_p_^2^ = 0.01, *p* = 0.5	*F*_(1,29)_ = 0.2, *η*_p_^2^ = 0.01, *p* = 0.7	*F*_(1,29)_ = 28.5, *η*_p_^2^ = 0.5, *p* < 0.001
Path length	*F*_(1,29)_ = 8.5, *η*_p_^2^ = 0.2, *p* = 0.007	*F*_(1,29)_ = 0.8, *η*_p_^2^ = 0.03, *p* = 0.4	*F*_(1,29)_ = 34.1, *η*_p_^2^ = 0.5, *p* < 0.001

F statistics with degrees of freedom, effect sizes of partial eta squared (*η*_p_^2^), and p values for the stance by group and stance by condition interactions, as well as stance main effect of CoP parameters. CoP, center of pressure; Norm., normalized; AP, anteroposterior; ML, mediolateral

Stance main effects were significant for trial duration, CoP displacement velocity, CoP SD and CoP displacement amplitude in anteroposterior and mediolateral directions, CoP area, and time-normalized CoP area (Tables 2, 3). Trial duration decreased from bipedal (60.0 s) to unipedal (43 ± 20 s) stance (*p* < 0.001), but did not differ between older adults with PD (49 ± 12 s) and older adults without PD (54 ± 7 s), or between firm (52 ± 10 s) and compliant (51 ± 10 s) surface conditions (*p* > 0.05), and all other variables increased from bipedal to unipedal stance. There were no group or condition main effects for sway variables; however, the group main effect for CoP SD mediolateral (*F*_(1,29)_ = 3.9, *η*_p_^2^ = 0.1, *p* = 0.06; Figure 3B) and condition main effect for anteroposterior CoP displacement amplitude (*F*_(1,29)_ = 3.2, *η*_p_^2^ = 0.1, *p* = 0.08) approached significance.

### 3.3. Electromyography

#### 3.3.1. Normalized EMG

There were no significant stance by group by condition interactions for normalized surface EMG of the plantar flexors, TA, or coactivation ratio. The stance by group interaction was significant for the LG (*F*_(1,29)_ = 6.3, *η*_p_^2^ = 0.2, *p* = 0.02) as EMG increased from bipedal (27.6 ± 21.9%) to unipedal (65.4 ± 37.8%) stance and older adults with PD had a greater increase compared to older adults without PD. Greater LG EMG in older adults with PD across both stances was reflected in the significant group main effect: *F*_(1,29)_ = 24.1, *η*_p_^2^ = 0.5, *p* < 0.001. From bipedal to unipedal stance, activation of the MG (*F*_(1,24)_ = 27.3, *η*_p_^2^ = 0.5, *p* < 0.001) and SOL (*F*_(1,27)_ = 58.0, *η*_p_^2^ = 0.7, *p* < 0.001) increased, but did not differ between older adults with PD and older adults without PD (MG: F_(1,24)_ = 2.4, *η*_p_^2^ = 0.09, *p* = 0.1; SOL: F_(1,27)_ = 0.04, *η*_p_^2^ = 0.001, *p* = 0.9). TA activation increased from bipedal to unipedal stance (*F*_(1,25)_ = 127.5, *η*_p_^2^ = 0.8, *p* < 0.001), and was greater in older adults with PD compared to older adults without PD (F_(1,25)_ = 6.2, η_p_^2^ = 0.2, *p* = 0.02). There was no stance by group (*F*_(1,20)_ = 0.02, *η*_p_^2^ = 0.001, *p* = 0.9) interaction, or main effects of stance (*F*_(1,20)_ = 2.0, *η*_p_^2^ = 0.09, *p* = 0.2), or group (*F*_(1,20)_ = 2.9, *η*_p_^2^ = 0.1, *p* = 0.1) for coactivation ratio. There were no condition main effects or interactions for surface EMG (Table 4).

**Table 4:** Normalized electromyography (EMG).

*Firm*	MG (%)	LG (%)	SOL (%)	TA (%)	Coactivation ratio (%)
Bipedal PD	20.5 ± 10.4	36.7 ± 25.9^#^	25.2 ± 10.9	23.1 ± 22.7^#^	32.1 ± 36.8
Bipedal OA	18.7 ± 16.4	16.4 ± 7.5	21.7 ± 21.3	10.3 ± 9.9	30.1 ± 39.2
Unipedal PD	68.4 ± 44.6^*^	85.0 ± 41.0^*#^	66.2 ± 29.8^*^	85.1 ± 44.9^*#^	63.2 ± 66.6
Unipedal OA	52.6 ± 41.0^*^	42.9 ± 19.1^*^	73.8 ± 44.6^*^	64.6 ± 31.3^*^	44.7 ± 16.1
*Compliant*					
Bipedal PD	17.7 ± 11.4	39.9 ± 27.6^#^	25.5 ± 10.3	29.7 ± 27.6^#^	65.3 ± 66.3
Bipedal OA	16.5 ± 7.2	16.1 ± 6.3	23.4 ± 19.8	10.1 ± 6.7	18.5 ± 11.8
Unipedal PD	72.4 ± 53.8^*^	92.9 ± 35.3^*#^	73.8 ± 44.6^*^	106.8 ± 62.7^*#^	58.0 ± 59.2^*^
Unipedal OA	37.2 ± 18.8^*^	38.3 ± 14.6^*^	80.1 ± 58.6^*^	54.1 ± 22.8^*^	33.0 ± 18.0^*^

Values are root mean squared EMG normalized to their respective maximum value during plantar flexion (MG, LG, SOL) or dorsiflexion (TA) MVC and expressed as percentages. Coactivation ratio was calculated by dividing the normalized TA activation by the sum of the normalized plantar flexors EMG and multiplying by 100. PD, older adults with Parkinson’s disease; OA, healthy older adults. MG, medial gastrocnemius; LG, lateral gastrocnemius; SOL, soleus; TA, tibialis anterior. *, differs from bipedal stance for same surface condition (*p* < 0.001). #, differs between older adults with PD and health older adults within same stance and condition (*p* < 0.05).

#### 3.3.2. Intermuscular coherence

**Figure 4:**
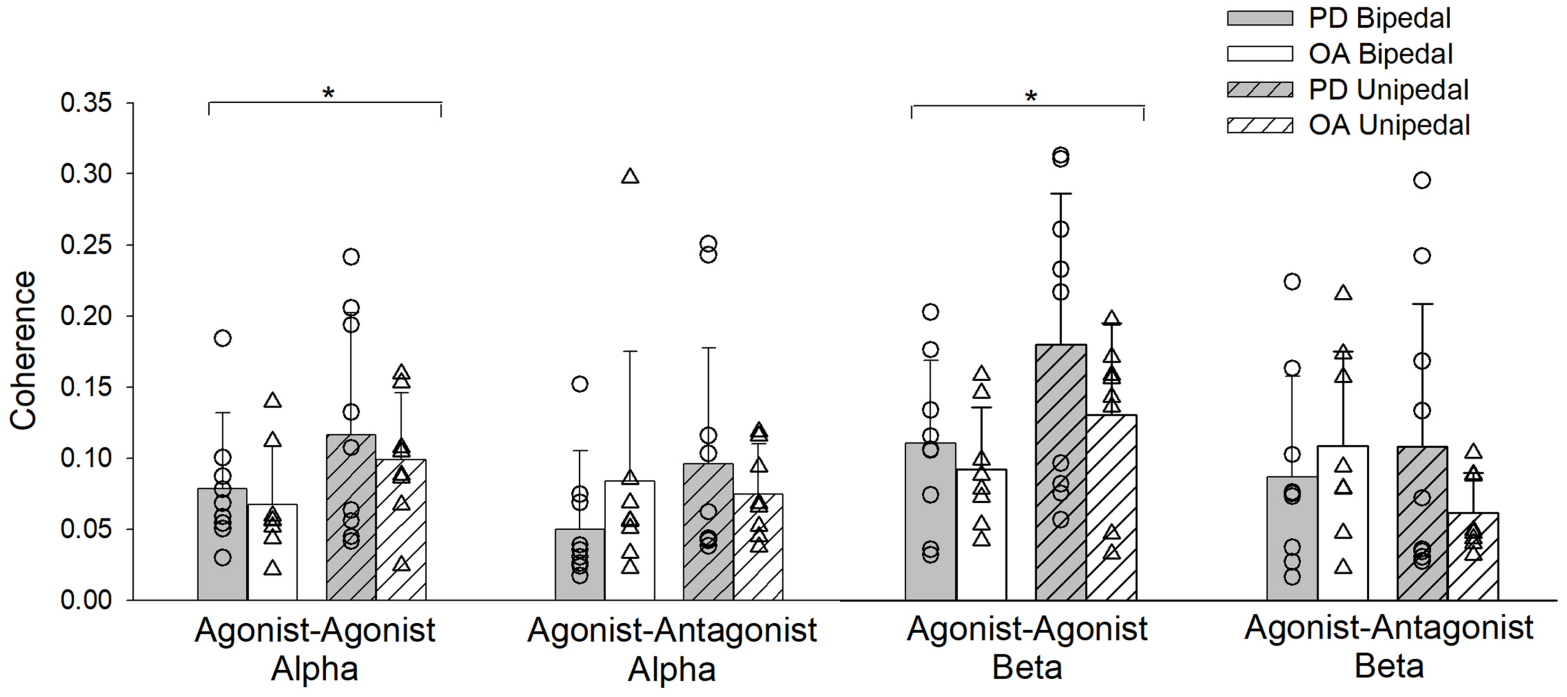
Alpha and Beta band coherence for pooled agonist–agonist and agonist–antagonist muscle pairs during bipedal (open bars) and unipedal stances (hatched bars) for older adults with Parkinson’s disease (PD, gray bars) and older adults without PD (OA, white bars). ^*^, bipedal differs from unipedal within the agonist–agonist alpha and beta coherence bands for older adults with PD and older adults without PD.

The stance by group by condition interaction for EMG coherence was not statistically significant for agonist–agonist and agonist–antagonist muscle pairs in both the alpha and beta bands. The stance by group and stance by condition interactions were also not significant for agonist–agonist alpha (*F*_(1,29)_ = 0.06, *η*_p_^2^ = 0.002, *p* = 0.8; *F*_(1,29)_ = 0.2, *η*_p_^2^ = 0.007, *p* = 0.7) and agonist–antagonist alpha (*F*_(1,29)_ = 3.5, *η*_p_^2^ = 0.1, *p* = 0.07; *F*_(1,29)_ = 0.1, *η*_p_^2^ = 0.004, *p* = 0.7) muscle pairs. Agonist–agonist alpha coherence increased from bipedal to unipedal stance (*F*_(1,29)_ = 6.6, *η*_p_^2^ = 0.2, *p* = 0.02), while agonist–antagonist alpha coherence did not differ between stances (*F*_(1,29)_ = 1.6, *η*_p_^2^ = 0.05, *p* = 0.2). Alpha band coherence did not differ between older adults with PD and older adults without PD (agonist–agonist alpha: *F*_(1,29)_ = 0.7, *η*_p_^2^ = 0.02, *p* = 0.4; agonist–antagonist alpha: *F*_(1,29)_ = 0.1, *η*_p_^2^ = 0.004, *p* = 0.7) or between firm and compliant conditions (agonist–agonist alpha: *F*_(1,29)_ = 0.03, *η*_p_^2^ = 0.001, *p* = 0.9; agonist–antagonist alpha: *F*_(1,29)_ = 0.02, *η*_p_^2^ = 0.001, *p* = 0.9; Figure 4).

There was a stance by group interaction for agonist–antagonist coherence in the beta frequency band (*F*_(1,29)_ = 6.7, *η*_p_^2^ = 0.2, *p* = 0.02), as going from bipedal to unipedal stance increased coherence in older adults with PD, but decreased for older adults without PD. However, *post-hoc* tests between groups were not statistically significant in the bipedal (*p* = 0.4) and unipedal (*p* = 0.08) stances (Figure 4). Beta band agonist–antagonist coherence did not differ between bipedal and unipedal stance (*F*_(1,29)_ = 0.9, *η*_p_^2^ = 0.03, *p* = 0.3), or between firm and compliant conditions (*F*_(1,29)_ = 0.2, *η*_p_^2^ = 0.006, *p* = 0.7). Agonist–agonist beta band coherence increased from bipedal to unipedal stance (*F*_(1,29)_ = 13.7, *η*_p_^2^ = 0.3, *p* < 0.001), but did not differ between groups (*F*_(1,29)_ = 2.4, *η*_p_^2^ = 0.08, *p* = 0.1) or conditions (*F*_(1,29)_ = 0.04, *η*_p_^2^ = 0.001, *p* = 0.9; Figure 4).

## 4. Discussion

We report comparisons of CoP displacement and lower limb intermuscular coherence in older adults with PD and non-Parkinsonian older adults during static balance in bipedal and unipedal stances on firm and compliant surfaces. In the current study, the older adults with PD were on dopaminergic medication, but still had greater SD of the CoP displacement in the mediolateral direction (*p* = 0.06), while the older adults without PD had significantly longer sway path lengths in unipedal stance that likely resulted from their 18% non-statistically greater postural sway amplitude in the anteroposterior axis. Older adults with PD had greater activation of the LG and TA muscles during the balance tasks, but coherence between agonist–agonist and agonist–antagonist muscle pairs did not differ between groups. Our data highlight that postural stability is altered in older adults with PD, and that further studies are required to better understand the progression of postural instability and interaction of drug therapy in neurologically compromised older adults.

The added task difficulty of going from bipedal to unipedal stance increased all CoP displacement parameters in both groups corroborating previous findings (Watanabe et al., 2018). However, unlike previous studies (Mitchell et al., 1995; Błaszczyk et al., 2007; Chastan et al., 2008; Kamieniarz et al., 2021), a greater area, range, or velocity of postural sway was not detected statistically for this group of older adults with PD compared to the age-matched older adults without PD. The variability (SD) of CoP in the mediolateral axis was 28% greater in older adults with PD compared to the older adults without PD, and this difference approached statistical significance at *p* = 0.06 with a medium-large effect size (*η*_p_^2^ = 0.1). Greater mediolateral CoP SD in individuals with PD has been suggested as a postural stabilization strategy early in the disease progression (Chastan et al., 2008), and this strategy was seemingly evident in our sample of older adults with PD.

The older adults without PD had longer path lengths compared to older adults with PD during unipedal stance. The longer path lengths in the older adults without PD may be indicative of a proposed exploratory behavior of postural sway, whereby the central nervous system uses CoP fluctuations to gain sensory feedback and minimize postural sway (Carpenter et al., 2010; Michaud et al., 2021). This exploratory mechanism may have aided the older adults without PD in minimizing their CoP area during the tasks. Shorter path lengths in older adults with PD are suggestive that this group is unable to use this exploratory strategy. Changes in CoP velocity have also been shown to provide information to the central nervous system indicating changes in direction and intensity of center of mass position (Masani et al., 2003). Smaller increases in CoP velocity from bipedal to unipedal stance in older adults with PD lends further support that they may be attempting to minimize increases in sway during unipedal stance and are not benefiting from added forms of feedback.

The lack of increased CoP displacement during the compliant surface condition was surprising, given that prior studies in healthy older adults have demonstrated increases in CoP velocity, anteroposterior and mediolateral CoP amplitude, and CoP SD during a foam condition compared to a no-foam or rigid force plate condition (Baudry et al., 2014; Papegaaij et al., 2014; Gosselin and Fagan, 2015). Although foam density is not always reported (Baudry et al., 2014; Papegaaij et al., 2014), the foams used by Gosselin and Fagan (2015) were denser (0.04–0.06 g/cm^3^) than the foam used in the present study (0.02 g/cm^3^). Thus, the lower density foam used here may not have provided a sufficient challenge relative to the firm surface condition to increase sway.

The added challenge of maintaining unipedal compared to bipedal stance required greater relative activation of the MG, LG, SOL, and TA by both groups, as was previously observed in young adults (García-Massó et al., 2016). Reduced strength in older adults with PD likely contributed to greater relative activation of LG and TA compared to the older adults without PD. The SOL and MG activation did not differ between groups, which may align with the age-related maintenance of neuromuscular properties of the SOL (Dalton et al., 2008), and our data may suggest that in older adults with PD, both the MG and SOL muscles are resistant to early neurological influence.

Increases in alpha and beta agonist–agonist coherence from bipedal to unipedal stance indicate that greater coordination between the MG, SOL and LG is occurring in order to execute the unipedal balance task. Surprisingly, agonist–antagonist coherence did not increase from bipedal to unipedal stance and suggests the frequency content of inputs to opposing muscle groups remained similar regardless of task difficulty, or that increasing coherence in the agonist–agonist muscle pairs is a core strategy used to compensate for the added instability of unipedal stance. Our findings of increased beta band agonist–agonist coherence in bipedal to unipedal stance align with a previous study in young and older adults (Watanabe et al., 2018), and this study extends the findings to the alpha band. Findings from magnetoencephalogram-EMG coherence during isometric upper limb contractions (Conway et al., 1995) indicate that beta band frequency originates from the primary motor area of the brain, and the increase from bipedal to unipedal stance we and others (Watanabe et al., 2018) have observed could be attributed to greater cortical involvement with increased task difficulty in both non-Parkinsonian older adults and those with PD. The underlying origin of increases in intermuscular coherence from bipedal to unipedal stance needs to be established. There is evidence linking beta band coherence to cortical sources (e.g., Conway et al., 1995), and contributions from stretch-reflex, motor cortex, brainstem, and cerebellum have been associated to adaptations in alpha band coherence (Laine and Valero-Cuevas, 2020), and thus peripheral and central systems are adapting to coordinate muscle activity in an effort to maintain postural stability in unipedal stance.

In this balance paradigm, intermuscular coherence did not differ between older adults with PD on dopaminergic treatment who had high motor function and age-matched older adults without PD. Treatment with deep brain stimulation increases upper-limb coherence in an isometric task in older adults with PD following withdrawal of levodopa medication (Marsden et al., 2001), and others have shown greater coherence in older adults with PD compared to non-Parkinsonian older adults for lower limb (Flood et al., 2019) and upper limb tasks (Laine and Valero-Cuevas, 2020) during the ON phase of levodopa treatment. Our data add to this existing literature by implying that during a balance task, intermuscular coherence might not be increased in older adults with PD who have high motor function and are tested during the ON phase of the levodopa cycle, and that lower-limb intermuscular coherence is not a contributor to increased postural instability. This finding aligns with clinical observations that postural instability is a less dopamine-responsive symptom, and aligns with the literature showing treatment may improve agonist–agonist intermuscular coherence (Marsden et al., 2001). The lack of observed group differences in intermuscular coherence may also be a result of our sample of PD having high motor function and not experiencing as large of a physiological response to the levodopa treatment as those with more severe disease progression (Marsden et al., 2001). This study is the first to examine plantar flexor and dorsiflexor intermuscular coherence in persons with PD during a balance task, and it is unknown if disease-related differences in intermuscular coherence are dependent on the type of task being performed. Future studies examining intermuscular coherence in older adults with PD should expand the coherence analysis to comparatives between upper and lower limbs, and for balance tasks other postural muscles such as the hip adductors and abductors. It has been shown that force steadiness of these muscle groups are predictive of postural sway area rate (Davis et al., 2020). The lack of group differences observed in our study could also be attributed to a small sample size, which reduces the power of the statistical comparisons. The use of balance paradigms is important for older adults with PD because of the risk of falls and injury, and we may see differences comparing treated to untreated states, but these tasks are difficult from a measurement standpoint as they cannot be maintained for long durations. The large variation in function and drug therapy with the progressive nature of PD likely contributed to the high variability seen within our data leading to minimal between-group differences. To further elucidate the role of intermuscular coherence on postural sway in older adults with PD, this should be studied in more advanced stages of disease progression both on and off treatment.

## 5. Conclusion

This study investigated intermuscular coherence of lower-leg muscles during bipedal and unipedal stance on firm and compliant surfaces in older adults with PD and older adults without PD. As task difficulty increased from bipedal to unipedal stance, agonist–agonist intermuscular coherence increased. The CoP path length was shorter in older adults with PD during unipedal stance, and they required greater activation of the LG and TA to perform the balance tasks. Our data demonstrate that there are minimal differences in postural sway between older adults with and without PD, and muscle activation is increased, but the coordination between the muscle activities—as measured with coherence—is unaltered at this stage of the disease. The effects of PD treatment and disease progression on the central coordination of muscle activity are of interest for future study to understand factors contributing to fall risk in older adults with PD.

## Data availability statement

The raw data supporting the conclusions of this article will be made available by the authors, without undue reservation.

## Ethics statement

The studies involving human participants were reviewed and approved by University of British Columbia Clinical Research Ethics Board. The patients/participants provided their written informed consent to participate in this study.

## Author contributions

RS collected and analyzed data and performed statistical analysis and writing of manuscript. AT designed MATLAB scripts used in analysis and contributed to analysis. OH analyzed data. SC designed MATLAB scripts used in analysis and assisted in coherence interpretation. BD: data interpretation and reviewing and editing of manuscript. DW: conceptualization of study and reviewing, and editing of manuscript. JJ: conceptualization of study, supervision, statistical evaluation and data interpretation, and reviewing and editing of manuscript. All authors contributed to the article and approved the submitted version.

## Funding

JJ and RS received support from the Natural Sciences and Engineering Research Council of Canada (NSERC; Grant number: 312038).

## Conflict of interest

The authors declare that the research was conducted in the absence of any commercial or financial relationships that could be construed as a potential conflict of interest.

## Publisher’s note

All claims expressed in this article are solely those of the authors and do not necessarily represent those of their affiliated organizations, or those of the publisher, the editors and the reviewers. Any product that may be evaluated in this article, or claim that may be made by its manufacturer, is not guaranteed or endorsed by the publisher.
